# Identification and Functional Analysis of Antifungal Immune Response Genes in *Drosophila*

**DOI:** 10.1371/journal.ppat.1000168

**Published:** 2008-10-03

**Authors:** Li Hua Jin, Jaewon Shim, Joon Sun Yoon, Byungil Kim, Jihyun Kim, Jeongsil Kim-Ha, Young-Joon Kim

**Affiliations:** 1 Department of Biochemistry, Yonsei University, Seoul, Korea; 2 Department of Molecular Biology, Sejong University, Seoul, Korea; University of California San Francisco, United States of America

## Abstract

Essential aspects of the innate immune response to microbial infection appear to be conserved between insects and mammals. Although signaling pathways that activate NF-κB during innate immune responses to various microorganisms have been studied in detail, regulatory mechanisms that control other immune responses to fungal infection require further investigation. To identify new *Drosophila* genes involved in antifungal immune responses, we selected genes known to be differentially regulated in SL2 cells by microbial cell wall components and tested their roles in antifungal defense using mutant flies. From 130 mutant lines, sixteen mutants exhibited increased sensitivity to fungal infection. Examination of their effects on defense against various types of bacteria and fungi revealed nine genes that are involved specifically in defense against fungal infection. All of these mutants displayed defects in phagocytosis or activation of antimicrobial peptide genes following infection. In some mutants, these immune deficiencies were attributed to defects in hemocyte development and differentiation, while other mutants showed specific defects in immune signaling required for humoral or cellular immune responses. Our results identify a new class of genes involved in antifungal immune responses in *Drosophila*.

## Author Summary

The innate immune response is the first line of defense against microbial infections in insects and mammals. In *Drosophila*, multiple defense mechanisms that contribute to the innate immune response include antimicrobial peptides (AMP), reactive oxygen species, phagocytosis and melanization. A search for genes involved in these immune processes identified sixteen mutants that exhibited increased lethality after infection. The diverse functions annotated to these genes indicate the complexity of the regulatory mechanisms required for defense against fungal infection. Lineage specific transcription factors and chromatin modifiers appeared to be required for proper development of functional hemocytes, while cytoskeletal regulators were required for phagocytotic activities of hemocytes. In addition, we identified several genes involved in the immune signaling required for AMP synthesis or melanization. These results may lay the foundation for defining a new class of genes that are involved in humoral and cellular antifungal immune responses.

## Introduction

Innate immunity is the first line of defense in multicellular organisms, and effectively prevents or limits infection after exposure to microbes [1]. The innate immune response to microbes triggers diverse humoral and cellular activities via signal transduction pathways that exhibit transphyletic conservation in animals [2]–[4]. In mammals, the adaptive immune system is recruited for complete elimination of microbes or microbial debris after initial neutralization or clearance by the innate immune system. However, *Drosophila* relies on humoral and cellular innate immune responses for protection against the barrage of microbes that thrive in its habitats [3]–[6].

A hallmark of the humoral response in *Drosophila* is the massive synthesis of antimicrobial peptides (AMPs) after immune challenge. AMPs are produced primarily by the fat body, the anatomical equivalent of the mammalian liver, and are secreted into the hemolymph where they directly kill invading microorganisms [6]. Genetic analysis has shown that AMP genes are regulated by various immunogenes through the Toll and Imd pathways [3],[6]. The Toll pathway is activated by both Gram-positive bacteria and fungi. Recognition of microbial components triggers proteolytic cleavage of the Toll ligand Spatzle (Spz) leading to activation of the Rel proteins, Dif and Dorsal [7]–[10]. In contrast, the Imd pathway mainly responds to Gram-negative bacteria and controls the expression of specific AMP genes by activating Relish [9],[11],[12].

In addition to strong antimicrobial activities provided by the humoral response, cell-mediated defenses also play an important role in the elimination of apoptosed cells and invading microbes or parasites [13]–[18]. The *Drosophila* hemocyte population consists of three cell types: plasmatocytes, crystal cells, and lamellocytes [19],[20]. Plasmatocytes represent 90–95% of all mature *Drosophila* hemocytes and function in the phagocytic removal of dead cells and microbial pathogens [15],[16]. Crystal cells, which constitute approximately 5% of the hemocyte population, are non-phagocytic cells that facilitate innate immune responses and promote wound healing through the process of melanization [15],[17],[21]. Lamellocytes are relatively large (15–40 µm), flat, adherent cells that facilitate the encapsulation and neutralization of objects too large to be engulfed by plasmatocytes [18]. These hemocytes are activated by microbial molecules through the same pattern recognition receptors as in the fat body, but the mechanisms leading to the activation of cellular immune responses are not fully understood.

Significant effort has focused on identifying components of the signaling pathways involved in regulating the innate immune response. Previous studies have identified a number of genes that are differentially regulated in hematocytes during microbial infection [22],[23]. However, the role of these genes in the immune response is only known for a few of them. To evaluate the role of these genes in antifungal immune responses, we examined the effect of individual mutations on the immune response of flies against *Beauveria bassiana* infection, and identified 16 mutants with increased sensitivity to *B. bassiana*. Examination of the sensitivities of these mutants to infection with several types of bacteria identified several mutants that were required mainly for defense against fungal infection. Examination of cellular immune responses revealed that transcription factors involved in chromatin remodeling or lineage specific differentiation were required for proper hemocyte development. Mutation of genes involved in cytoskeletal remodeling caused a strong defect in phagocytosis, while Trx-2 and DDB1 were required for development of functional crystal cells. The screen also identified several novel genes required for activation of antimicrobial peptide genes, indicating their involvement in signaling during pathogen specific immune responses. The distinct requirement of these genes for defense against different microbial infections also reveals the complexity of innate immune responses designed to compete with diverse offensive mechanisms used by microbes. In this paper, we present new findings on the regulation of cellular and humoral immune responses of *Drosophila* against fungal infection.

## Results

### Screening of immune defective mutant flies

**Figure 1:**
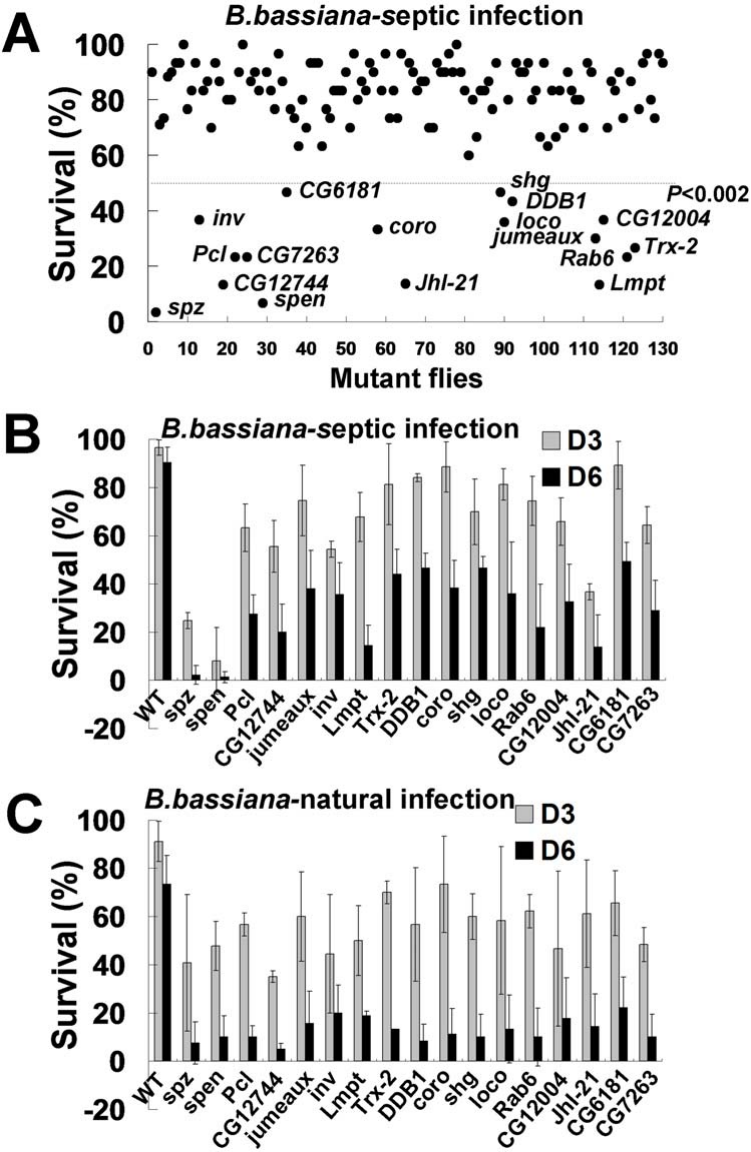
Screening of immune defective mutant flies. (A) The survival rate on day six after *Beauveria bassiana* (*B. bassiana*) septic infection is shown for the 130 mutant lines examined. Of 130 mutant lines, 16 had dramatically reduced resistance to *B. bassiana* infection, with a survival rate of less than 50% after six days (*p*<0.002). (B, C) Survival rate kinetics from three independent experiments are shown for the 16 mutants with increased sensitivity, (B) septic infection (C) natural infection. The *spz^rm7^* mutant was used as a positive control for *B. bassiana* infection. WT is wild type, and D3 and D6 stands for the survival at day 3 and day 6, respectively, post-infection.

Previously we identified genes that were differentially induced in SL2 cells after treatment with LPS/PGN or curdlan using *Drosophila* cDNA microarrays [24]. These LPS/PGN-or curdlan-induced genes are probably involved in diverse immune responses, such as activation of signaling pathways downstream of pathogen associated molecular pattern recognition receptors, induction of phagocytosis, and differentiation into a specialized immune effector cell type. Because these immune responses require crosstalk between different cell types in a physiological condition, expression profile analysis of SL2 cells alone may not provide a complete picture of gene regulation during infection. However, because SL2 cells display important characteristics of macrophages in an in vitro assay, we assumed that their expression pattern may reflect regulatory mechanism underlying some immune responses of macrophages. To identify key regulators of innate immunity, we obtained mutants of the genes that are differentially regulated following treatment with microbial components, and monitored their requirement for defense against infection. Out of 5,405 genes screened on the microarray, 231 and 1,151 genes were induced more than 1.6 fold after the LPS/PGN or curdlan treatment of SL2 cells, respectively. A search for congenic EP (Enhancer-Promoter) lines in which these differentially regulated genes were disrupted by a *P*-element insertion identified 130 lines (110 and 20 lines with a *P*-element inserted at the untranslated and coding regions of the differentially regulated genes, respectively) from the GenExel library (Daejeon, Korea). The *P*-element insertion positions of all the GenExel EP lines were confirmed twice independently by direct sequencing of the inverse PCR fragment amplified with *P*-element specific primers (data not shown). These results suggest that most of the defects associated with the EP lines are related to disruption of the candidate genes. About one-third (47 lines) of the EP lines obtained were homozygous lethal, indicating that the *P*-element insertion effectively disrupted function of the target genes. None of the 83 homozygote viable EP lines showed obvious developmental abnormalities. These results indicated that the EP lines could be used to screen for genes involved specifically in defense against microbial infection. Therefore, adult homozygote flies were screened for survival after infection with entomopathogenic fungi (*B. bassiana*) (Table S1). Although a developmental defect caused by heterozygocity of a gene is rare, functional insufficiency of a heterozygote is often observed under strong environmental stress such as infection, and can influence survival of the heterozygotes as shown in the study of *Dif ^1^* heterozygotes [8]. Based on this assumption, adult heterozygote flies were monitored for survival after fungal infection in the case of the homozygous lethal lines. To identify EP lines with a compromised defense against fungal infection, 30 adult flies from each of the 130 lines were pricked on the leg disc with a needle dipped into a concentrated solution of live *B. bassiana*, and the survival rate was followed over a six day period at 25°C. The septic infection with *B. bassiana* resulted in approximately 10% mortality in the wild type flies. Under the same infection conditions, most of the mutant flies showed similar levels of survival (Figure 1A, Table S1). However, 16 mutant lines, including six heterozygote flies (*Pcl*, *DDB1*, *shg*, *Rab6*, *CG6181*, *and CG7263*), were significantly more sensitive to fungal infection (*p*<0.002) (Figure 1A). In these cases, death was clearly associated with uncontrolled fungal growth, as the dead flies were covered with fungal hyphae (Figure S1).

To confirm the defects of the 16 lines, we first compared their survival rates after fungi infection with wild type and *spz* mutant as negative and positive controls, respectively, in three independent experiments. The repeated experiments revealed that the 16 EP lines had a clear defect in survival (Figure 1B). We next examined the survival rates after natural infection with *B. bassiana* to rule out the possibility that reduced viability resulted from septic injury rather than from fungal infection. When the flies were raised after being covered with spores for 1 min, the 16 mutant lines showed remarkably less survival comparable to that of the *spz* mutant, while wild type showed only a minor decrease in survival (Figure 1C). This result indicated that we have identified *Drosophila* mutants that have a reduced ability to defend against *B. bassiana* infection.

### Rescue of the mutant phenotype by precise *P*-element excision or by overexpressing the disrupted genes from an EP promoter

**Figure 2:**
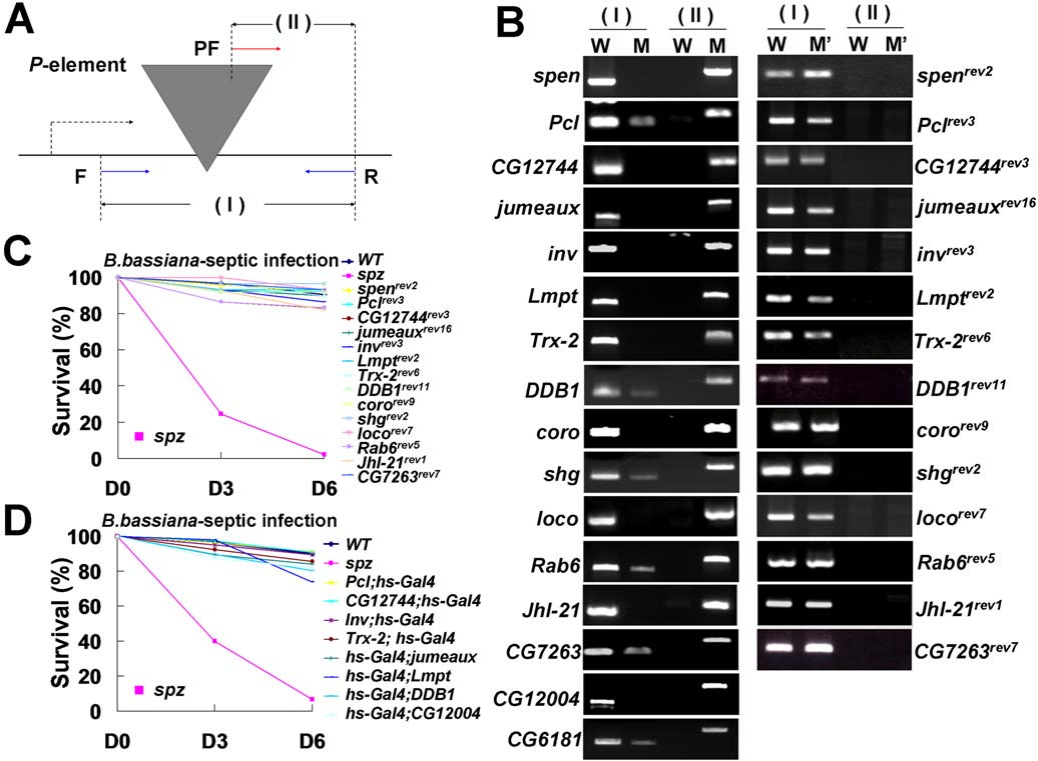
Confirmation of precise excision lines from *P*-element inserted mutant flies. (A) Relative positions and orientations of PCR primers used to confirm the position of *P*-element insertions and their precise excision. Primers F and R are complementary to the genomic DNA surrounding each *P*-element insertion site, while the PF primer is complementary to the *P*-element. PCR fragments amplified by the gene-specific primers (F and R) or by the *P*-element- and gene-specific primers (PF and R) are indicated as (I) and (II), respectively. (B) PCR fragments (I) and (II) amplified from wild type (W) or mutant (M) flies are shown in the left panel. The right panel shows corresponding PCR fragments amplified from the precise excision lines (M') of each mutant. Names of the specific precise excision alleles used in the analysis are indicated at the right. Survival kinetics of the precise excision lines (C) and the flies overexpressing wild type transgenes in mutant background (D). The *spz^rm7^* mutant was used as a positive control for *B. bassiana* infection.

To confirm that the increased sensitivity of these mutants to fungal infection is caused by specific disruption of the candidate genes by the *P*-element, we excised the *P*-element from the mutant flies by crossing with *P*[*ry* ^+^Δ2–3](99B)*Sb*/*TM6B*, *TB*. After excising the *P*-element from the germ cells, white-eye progeny were established as homozygous lines for all mutants. Excision of the *P*-element in each line was confirmed by PCR with primers specific to one end of the *P*-element (PF) and to target sequences surrounding the *P*-element insertion sites (F and R) (Figure 2A). The PF and R primer pairs amplified specific fragments (fragment II) from the *P*-element mutant lines confirming the mutation sites, but failed to amplify this fragment in any of the excised lines. On the other hand, F and R primer pairs that amplify the undisrupted target gene sequences (fragment I) failed to amplify specific fragments from the homozygous mutant flies and produced reduced levels of the amplification products from the heterozygous mutants (*Pcl*, *DDB1*, *shg*, *Rab6*, *CG6181*, *and CG7263*). These PCR primers specifically amplified products from all of the excised lines (Figure 2B). To confirm that these excised lines did not contain a small deletion or insertion at the *P*-element insertion sites, we cloned fragment I amplified from each excision line and sequenced them together with fragment II obtained from the corresponding *P*-element insertion mutants. This sequencing analysis confirmed that precise excision lines had been obtained for all mutants except *CG12004* and *CG6181* (data not shown).

After obtaining the precise excision lines for all mutants, we examined whether excision of the *P*-element from the mutants could revert their sensitivity to fungal infection to that of wild type flies. As shown in Figure 2C, similar survival rates (90%) were observed in both the wild type and the precise excision homozygous lines following fungal infection that caused complete death of the *spz* mutant (Figure 2C).

In addition to rescuing the increased lethality following infection by precise excision of the *P*-element, we tested whether overexpression of disrupted genes with the EP promoter inserted in front of the coding region could reverse the mutant phenotype. Half of the mutants contained a Gal4-dependent promoter (EP element) at the 5′ UTR in a forward orientation to the disrupted gene. Heat shock in combination with an *hs-Gal4* driver induced overexpression of the disrupted gene in this half of the EP mutant lines (Table S2). Thus, we generated flies carrying a copy of *hs-Gal4* driver and homo- or heterozygous *P*-element insertions, depending on the corresponding mutant configuration used for the screen. Quantitative RT-PCR analysis of the mutant EP lines revealed that the disrupted gene transcript was significantly less than that in the wild type. However, heat shock treatment (1 h at 37°C) in the presence of the *hs-Gal4* driver activated transcription of the target genes above the level observed in wild type flies (Figure S2). Consistent with this observation, lethality of the mutant lines reverted completely to wild type levels (Figure 2D). These results demonstrate that the genes identified from our screen are required for *Drosophila* antifungal immunity.

These genes identified in our screen encode proteins from many different functional classes including transcription factors involved in chromatin remodeling or lineage specific transcription (*spen*, *Pcl*, *CG12744*, *jumeaux*, *inv*, *and Lmpt*), cytoskeletal regulation (*coro*, *shg*, *loco*, *and Rab6*), DNA fragmentation, apoptosis and redox signaling (*CG7263*, *DDB1 and Trx-2*), along with a few genes (*CG6181*, *CG12004 and JhI-21*) of unknown function. Therefore, genes involved in immune responses ranging from development to cell movement were identified in this fungal defense screen.

### Specificity of genes for antifungal defense

**Figure 3:**
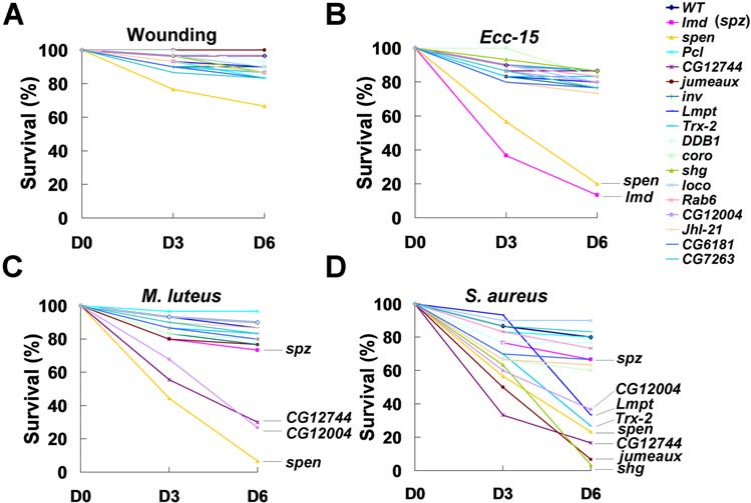
Survival rates of wild type and mutant flies following bacterial infection. (A). Survival rate kinetics for the 16 mutants with respect to wound healing. (B, C, D) Survival rates of the 16 mutants following septic infection with bacteria are shown, *Ecc-15* (Gram-negative) (B), *M. luteus* (Gram-positive) (C), and *S. aureus* (Gram-positive) (D). *Imd* and *spz* mutants were used to control for sensitivity to bacterial infections. The color code of each line is the same as in right side of figure.

To determine whether reduced survival of the mutant flies resulted from defective immune responses specifically to fungal infection, we examined the effect of these mutations on wound healing and defense against bacterial infection. When mutant flies were pricked with a sterile tungsten needle, the majority of the flies survived the wounding and only the *spen* mutant showed a minor decrease in survival (Figure 3A), suggesting that the reduced survival rates of these mutants, except *spen*, were caused by a defective defense against microbial infection. Septic infection with Gram-negative bacteria does not normally affect the viability of wild type flies. However, loss of a major antibacterial gene, such as *imd*, severely reduces survival following infection with Gram-negative bacteria. When the mutant flies were tested for susceptibility to *Ecc-15* infection, most showed no significant defect in survival. However, *spen* and *imd* mutants were highly sensitive to infection. Interestingly, *imd* was not required for defense against *Micrococcus luteus* (Gram-positive bacteria) [9],[25]. On the other hand loss of *spz* caused a minor defect in immune response against *M. luteus* infection as was shown [9],[26]. Similar infection analysis with *M. luteus* showed significantly more lethality in *spen*, *CG12744*, and *CG12004* than in *spz* mutants without affecting survival in most of the other mutants (Figure 3B, 3C). These results indicate that most of the genes except *spen* are not required to defend against Gram-negative bacteria, while two novel genes (*CG122744* and *CG1200*4) are required to defend against Gram-positive bacterial infection. We also tested survival of the mutant flies after *Staphylococcus aureus* infection. In addition to the three mutants susceptible to *M. luteus* infection, *jumeaux*, *Lmpt*, *shg*, and *Trx-2* mutants were highly susceptible to *S. aureus* infection (Figure 3D). This result indicated that more sophisticated immune responses are required to control the highly pathogenic *S. aureus*. Therefore, of the 16 genes found to be essential for anti-fungal defense, *spen* appears to be required for general immune responses, while nine genes (*Pcl*, *inv*, *DDB1*, *coro*, *loco*, *Rab6*, *JhI-21*, *CG6181*, and *CG7263*) are specifically required for anti-fungal defense. The other six genes (*CG12744*, *jumeaux*, *Lmpt*, *Trx-2*, *shg*, and *CG12004*) are differentially required, to defend against Gram-positive bacteria, depending on the pathogenic activities of the infecting bacteria. Because flies utilize several defense mechanisms against microbial infection, Gram-negative bacteria may be easily cured even if some mechanisms are not functional, while both cellular and humoral defenses may be needed to eradicate highly pathogenic microbes such as *S. aureus* and fungi.

### Effects on antimicrobial peptide gene expression

**Figure 4:**
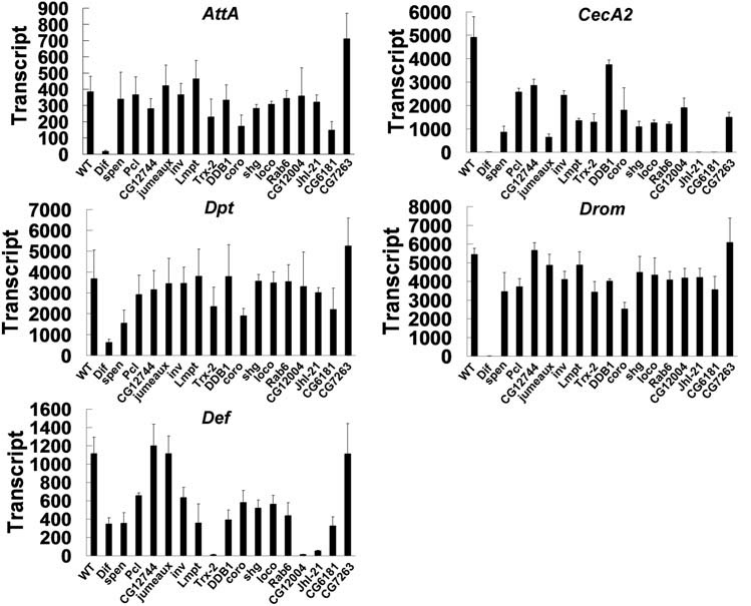
Expression profiles of antimicrobial peptide genes in wild type and mutant flies. Quantitative real time PCR analysis of antimicrobial peptide genes is presented. Wild type and mutant adult flies were infected with live spores of *B. bassiana*, total RNA was isolated from adult flies, and subjected to real time PCR analysis six hours after injection. The amount of transcripts in each sample were normalized to *RpL32* transcripts (AMP transcripts = normalized target mRNA expression in sample×1000). The mean and standard deviation of three independent experiments are shown. *AttA*, *Attacin A*; *CecA2*, *Cecropin A2*; *Drom*, *Drosomycin*; *Dpt*, *Diptericin*; *Def*, *Defensin*.

To determine whether the immune response was defective in each mutant, particularly in adults, we first examined the synthesis of diverse antimicrobial peptides (AMPs) in response to fungal infection. Quantitative RT-PCR analysis of five major AMP transcripts (*AttA*, *CecA2*, *Dpt*, *Drom*, and *Def*) revealed very low AMP transcript levels that are comparable to those in wild type flies prior to fungal infection in all of the mutants, indicating no major defect in the regulation of basal AMP expression in the mutants (data not shown). When the flies were challenged with fungal spores, all the five AMP genes were highly induced in wild type flies, and the expression of these genes was strongly reduced or abolished by mutation of the Toll-dependent transcription factor, *Dif*. Under the same infection condition, most of the mutant flies were defective in activation of certain types of AMP gene expression, and different AMP genes appear to require different genes for their activation in response to fungal infection (Figure 4). *AttA*, *Drom*, and *Dpt* synthesis in response to fungal infection was not affected in most of the mutants. However, mutations in *Trx-2*, *coro*, *CG6181* and *spen* caused moderate defects in their activation. In contrast, the induction of *CecA2* and *Def* by fungal infection was significantly reduced in most of the mutants analyzed. *CecA2* expression was defective in most of the mutants except *DDB1*. In particular, *CecA2* expression was completely abolished in *JhI-21* and *CG6181* mutants, and was highly repressed in *spen* and *jumeaux* mutants. Activation of *Def* expression was affected in most of the mutants except *CG12744*, *jumeaux*, and *CG7263*, with the most severe defects found in *Trx-2*, *CG12004*, and *JhI-21* mutants. Therefore, *spen*, *Trx-2*, *coro*, and *CG6181* appear to be required to activate most of the antimicrobial peptide genes upon fungal infection, while *CG12004* and *JhI-21* appear to be required to activate *Def* and *CecA2*, respectively. However, *DDB1* does not seem to be required to activate AMP expression induced by fungal infection.

### *In vivo* assessment of phagocytosis

**Figure 5:**
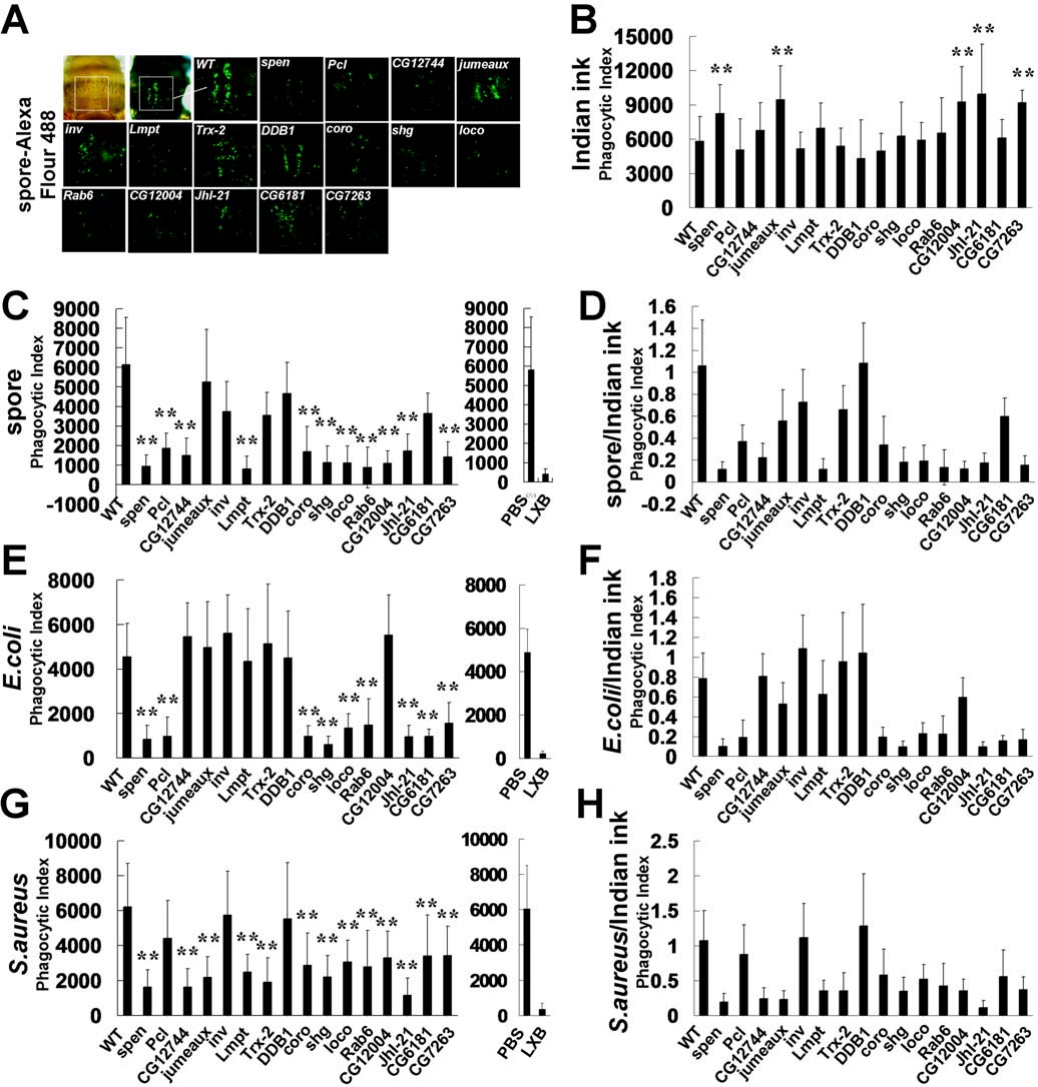
*In vivo* phagocytosis in adult flies. (A) Adult males of the indicated genotypes were injected with Alexa Fluor 488-labeled heat killed spores of *B. bassiana*. (B, C, E, G) Quantitation of in vivo phagocytosis of India ink, spore and bacteria. (B) India ink, (C) Alexa Fluor 488-labeled heat killed spores of *B. bassiana*, (E) Fluorescein conjugated *E. coli* (K-12), (G) Fluorescein conjugated *S. aureus*. Phagocytosed signals were observed under a Zeiss Axioplan 2 microscope. Phagocytic index was derived by multiplying the area of the India ink and fluorescence signal measured. Phagocytosis was inhibited by prior injection of latex beads in wild type. LXB, CML latex beads. (D, F, H) A phagocytic index was obtained by multiplying phagocytosing signals with the mean area of internalized India ink. (D) spore, (F) *E. coli*, (H) *S. aureus*. The mean and standard deviation of 10–16 adult flies were analyzed for each genotype. *p*-values were calculated by Student's t-test. India ink, **p<0.1. Fungi and bacteria, **p<0.007.

Along with the humoral response, which is mediated mainly by the synthesis of specific antimicrobial peptides, the phagocytosis of invading microbes by hemocytes is another major defense mechanism of adult flies. Hemocytes are mostly sessile and cannot easily be removed from adult flies. However, these cells can be observed through the cuticle, and clusters of hemocytes are present under the dorsal surface of the abdomen, along the dorsal vessel [27],[28]. To assay the phagocytic activities of mutant hemocytes in vivo, wild type and mutant adult male flies were infected with Alexa Fluor 488-labeled spores of *B. bassiana*, and the level of fluorescence from phagocytosed spores was measured after quenching the signal from spores outside the hemocytes (Figure 5, A and C). Wild type flies showed a strong fluorescence signal from the phagocytosed spores; however, eleven (*spen*, *Pcl*, *CG12744*, *Lmpt*, *coro*, *shg*, *loco*, *Rab6*, *CG12004*, *JhI-21*, *and CG7263*) of the sixteen EP mutants had a weak fluorescence signal, indicating that the mutant hemocytes were defective in uptake of the spores. To determine whether the reduction in phagocytosed spores in some of the mutant flies resulted from the reduced hemocytes, we measured the number of hemocytes present under the dorsal surface of the abdomen of each of the mutant flies. Hemocytes were visualized by injecting India ink, and the amount of black particles taken up by each mutant hemocyte was quantified. India ink staining revealed that most of the mutants contained hemocytes that were comparable to or even higher (*spen*, *jumeaux*, *CG12004*, *JhI-21*, *and CG7263*) than wild type (Figure 5B). Therefore, the reduced fluorescent signals appear to reflect defective phagocytosis rather than fewer hemocytes in the mutants. When the fluorescent signal of the phagocytosis assay was normalized to the number of hemocytes estimated by India ink staining, we observed a moderate defect in *jumeaux* mutant in addition to the eleven mutants that showed clear phagocytic defects (Figure 5D). In addition, these fluorescent signals appeared to depend on the phagocytotic machinery of the hemocytes since injection of excessive latex beads competed out the signal completely (Figure 5C). Therefore, in addition to obvious phagocytotic components (cytoskeletal regulators; *coro*, *shg*, *loco*, and *rab6*), genes in diverse categories, such as transcription factors (*spen*, *Pcl*, *CG12744*, and *Lmpt*), cell death regulators (*CG7263*), and other novel factors (*CG12004* and *JhI-21*), appear to be required to phagocytose fungal spores.

We next examined whether similar genes are required to phagocytose bacteria. The *E. coli* phagocytosis signal was strongly reduced in flies carrying a mutation in the cytoskeletal regulators (*coro*, *shg*, *loco*, and *rab6*) or in some of the genes required to phagocytose fungal spores (*spen*, *Pcl*, *JhI-21*, *and CG7263*). In addition, *CG6181* appeared to be required specifically for *E. coli* phagocytosis (Figure 5, E and F). In addition to genes required to phagocytose *E. coli*, phagocytosis of *S. aureus* requires additional genes that function as transcription factors (*CG12744*, *jumeaux*, and *Lmpt*) or as a redox regulator (*Trx-2*) (Figure 5G, 5H). These results indicate that genes involved in cytoskeletal and cell death regulation, along with *spen* (chromatin regulator) and *JhI-21* (transporter induced by juvenile hormone), are generally required for phagocytosis of diverse microorganisms. In contrast, *jumeaux* and *Trx-2* are required to specifically phagocytose *S. aureus*, which is known to utilize diverse immune evading mechanisms [29]–[31]. Therefore, hemocytes appear to require genes involved in diverse cellular functions to mediate a proper cellular immune response against fungal and bacterial infection.

### Analysis of *Drosophila* larval hematopoiesis

**Figure 6:**
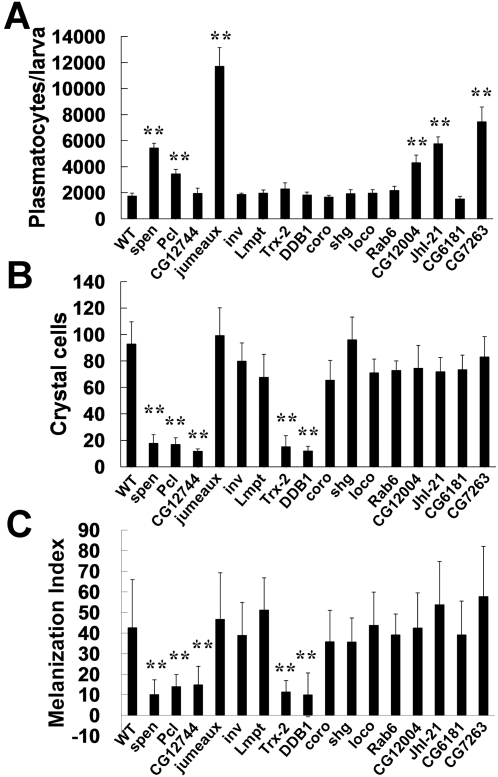
Analysis of *Drosophila* larval hematopoiesis. (A) Analysis of circulating plasmatocytes. Plasmatocytes were counted from at least six third instar larvae of each genotype; error bars represent the SD of the mean from 3–6 independent experiments. (B, C) Analysis of crystal cell development. (B) Third instar larvae were heated to 60°C for 10 min in a water bath to visualize crystal cells through the cuticle. Crystal cell counts from the sessile population of the last two posterior dorsal segments of third instar larvae of the indicated genotype are shown. (C) Third instar larvae pricked with a clean standard needle led to a hemocoelic melanization reaction. Melanization index was derived by the area with the mean intensity of melanization signal measured. 12–20 larvae were analyzed for each genotype, error bars represent standard deviation. In each panel, *p*-values were calculated by Student's t-test. **p<0.01.

The analysis of hemocytes in adult flies revealed that some mutants are defective in the activation of both phagocytosis and AMP synthesis, and showed an abnormal number of hemocytes. This observation suggested that some of the immune defects were caused by inappropriate hematopoiesis. To test this idea we examined whether hemocyte development in these mutants occurred normally. We first compared the number of circulating plasmatocytes in third instar larvae of mutant and wild type flies. Since the number of circulating hemocytes increases rapidly during development, we staged the wandering larvae according to the presence or absence of food in the gut [32]. Because the mutants showed no obvious developmental defects or delay, this method enabled us to measure the circulating hemocytes of each mutant at a comparable developmental stage. However, we cannot rule out the possibility that hemocyte development in certain mutant larvae was affected in some degree by the mutations. When we counted the circulating hemocytes, late third instar larvae of six mutants (*spen*, *Pcl*, *jumeaux*, *CG12004*, *JhI-21*, *and CG7263*) displayed a 2- to 6-fold increase in the number of plasmatocytes (Figure 6A), which is consistent with the higher number of hemocytes observed in the adult of the same mutants. Therefore, the defective immune responses observed in many mutants appear to be related to abnormal plasmatocyte proliferation.

The defects in plasmatocyte proliferation in some of the immune compromised mutant flies prompted us to examine the effect of the mutations on crystal cell development. To measure the number of crystal cells in the larvae of each mutant, third instar larvae were heated to 60°C for 10 min to induce blackening of mature crystal cells. *spen*, *Pcl*, *CG12744*, *Trx-2*, *and DDB1* mutant larvae showed fewer crystal cells than did wild type larvae (Figure 6B). We also tested the functional activity of crystal cells in each mutant by injuring third instar larvae with a clean needle and measuring the level of melanization in each mutant larva. Strong melanization at the injury site was observed in wild type larvae and most of the mutant larvae. However, *spen*, *Pcl*, *CG12744*, *Trx-2*, *and DDB1* mutant larvae showed much less melanization induced by injury, consistent with their defects in crystal cell proliferation (Figure 6C, Figure S3). Therefore, *spen* and *Pcl*, which are involved in chromatin regulation, appear to function in the development of both plasmocytes and crystal cells. It is intriguing that genes involved in the recognition of damaged DNA (*DDB1*), redox regulation (*Trx-2*), and a novel transcription factor (*CG12744*) are also required for proper crystal cell development.

## Discussion

The immune system employs multiple layers of defense against pathogens and it is difficult for most invading bacteria to overcome these redundant host defense barriers. However, fungi are largely opportunists, causing infection when any of host defenses are breached. *Beauveria bassiana* is an entomopathogenic fungus that causes a disease in insects known as white muscadine disease. Unlike bacterial pathogens, once inside the insect it produces a toxin that weakens the host immune system. To search for important factors within the entire *Drosophila* immune system that are required for antifungal defense, we screened for genes specifically required for survival following *B. bassiana* infection and identified several genes involved in diverse aspects of cellular and humoral immune responses (summarized in Table 1). Although some of the mutants showed general immune defects and were susceptible to both fungal and bacterial infection, most of the other mutants exhibited distinct immune defects and were susceptible only to fungal or to highly pathogenic bacterial infection. This increased susceptibility specifically to fungal infection might result from defects in defenses against fungal-specific pathogenic molecules, but it is also possible that anti-fungal responses require more diverse immune defense mechanisms than bacterial infection, such that mutants with specific defects could overcome bacterial infection using other functional immune responses.

**Table 1:** Summary of the cellular and humoral immune responses of the sixteen mutants.

	*spen*	*Pcl*	*CG12744*	*jumeaux*	*inv*	*Lmpt*	*Trx-2*	*DDB1*	*coro*	*shg*	*loco*	*Rab6*	*CG12004*	*JhI-21*	*CG6181*	*CG7263*
**Survival**																
Fungi	---	---	---	---	---	---	---	---	---	---	---	---	---	---	---	---
*Ecc-15*	---	+	+	+	+	+	+	+	+	+	+	+	+	+	+	+
*M. luteus*	---	+	---	+	+	+	+	+	+	+	+	+	---	+	+	+
*S.aureus*	---	+	---	---	+	---	---	+	+	---	+	+	---	+	+	+
**Phagocytosis**																
*Fungi*	---	---	---	+	+	---	+	+	---	---	---	---	---	---	+	---
*E.coli*	---	---	+	+	+	+	+	+	---	---	---	---	+	---	---	---
*S.aureus*	---	+	---	---	+	---	---	+	---	---	---	---	---	---	---	---
**Proliferation**																
Plasmatocytes	---	---	+	---	+	+	+	+	+	+	+	+	---	---	+	---
Crystal cells	---	---	---	+	+	+	---	---	+	+	+	+	+	+	+	+
**AMP genes**																
*AttA*	+	+	+	+	+	+	-	+	-	+	+	+	+	+	-	+
*CecA2*	-	-	-	-	-	-	-	+	-	-	-	-	-	---	---	-
*Dpt*	-	+	+	+	+	+	+	+	-	+	+	+	+	+	+	+
*Drom*	+	+	+	+	+	+	+	+	-	+	+	+	+	+	+	+
*Def*	-	-	+	+	-	-	---	-	-	-	-	-	---	---	-	+

---, severe defects; -, defect; +, no defect.

Inappropriate development of plasmocytes and crystal cells appears to be one of the main causes of the immune defects in the mutants identified in this screen. Spen and Pcl play essential roles in the chromatin modification needed for hemocyte development [33]–[35]. Mutations in these genes must prevent progenitor hemocytes from differentiating into functional plasmocytes or crystal cells, and cause pleiotrophic defects in diverse aspects of immune function. Pcl appears to be less important for bacterial infection than does Spen, but the difference may be due to different degrees of gene inactivation in the *Pcl* heterozygotes vs. *spen* homozygotes, rather than from differences in regulatory function. A similar explanation could be applied to fungal specific defects of the other heterozygote mutants.

In addition to chromatin regulators, it is intriguing that CG12744 and Jumeaux are required specifically for the development of crystal cells and plasmocytes, respectively. CG12744 is a novel transcription factor; in contrast, Jumeaux is a transcription factor expressed in embryonic CNS and is required in neuronal development [36]. How these transcription factors regulate the development of specific hemocytes is not known, but their expression pattern and requirement in a specific blood cell type suggest a role in the maturation of distinct types of hemocytes.

Crystal cell differentiation also requires Trx-2 (thoredoxin-2) and DDB1 (Damaged DNA Binding protein 1). Trx-2 regulates redox signaling, which is essential for the activation of immune effector functions [30],[31],[37] and the melanization reaction. The misregulation of redox signals by the loss of Trx-2 may affect early steps in the signal transduction pathway induced by pathogen recognition, causing diverse defects in immune function. DDB1 is involved in the recognition of damaged DNA in dying cells or in invading pathogens and is required for plasmocyte development [38]. However, how DDB1 affects crystal cell function is not known.

In addition to transcription factors, cytoskeletal regulators are another major group of genes required to defend against infection. Coro has F-actin binding activity and is required for membrane trafficking [39]. Shg is a *Drosophila* Cadherin and is required for cell motility and adhesion [40],[41]. Loco and Rab6 are involved in asymmetric cell division and vesicle transport, respectively [42],[43]. Therefore, these proteins must be required for cytoskeletal rearrangement during phagocytosis. It is interesting that these mutants also showed defects in AMP synthesis. Efficient recognition of pathogens or subsequent signaling may require cytoskeletal rearrangement.

We also identified several novel genes, whose function in innate immunity has not been previously suggested. CG12004 is a novel protein without known protein motifs, but it appears to play an important role in plasmocyte development. JhI-21 is a cationic amino acid transporter induced by juvenile hormone [44]. It is required for plasmocyte development and affects their phagocytosis and AMP synthesis. CG6181 is a novel protein and CG7263 are known to be involved in apoptosis [45]. Recently, endocytic degradation by apoptosis was suggested to play essential roles in defense against pathogenic microbes that can escape from endosomes to cytoplasm [46].

Several novel genes identified from this screen appear to have essential roles in defense against both fungi and bacteria, indicating their roles in the regulation of primary immune responses. The putative functions of these newly identified genes (Spen, CG12744, Jumeaux, Lmpt, Trx-2, Shg, and CG12004) as transcription factors, redox regulator, or cell adhesion molecule hints at their role in regulating immune responses. Therefore, further study of these genes will provide important insight into regulatory mechanism of the *Drosophila* immune system.

Our results showed that complex immune reactions are required to defend against fungal infection in *Drosophila*, and identified key regulatory components involved in these immune reactions. These findings increase our understanding of the mechanisms underlying cellular and humoral aspects of *Drosophila* antifungal immunity, and have significant implications in the treatment of human diseases caused by fungi.

## Materials and Methods

### *Drosophila* stocks

*Drosophila melanogaster* strains were cultured on a standard cornmeal-yeast medium at 25°C and 60% humidity. Mutant flies containing a *P*-element at the translated/untranslated region of the candidate genes (Table S1) were purchased from GenExel (Daejeon Korea). Because the GenExel EP lines contain Gal4 binding sites, overexpression of Gal4 can induce strong expression of adjacent endogenous genes in which an EP element is inserted at the 5′ UTR in a forward orientation [47]. To activate transcription of *P*-element inserted genes from the EP promoter, we crossed mutant flies containing a *P*-element at the 5′ UTR in a forward orientation with *hs-Gal4* driver (Bloomington Stock Center). For homozygous viable lines, we generated flies carrying a homozygous *P*-element inserted chromosome in addition to a *hs-Gal4* driver. Overexpression of target genes was achieved by heat shocking the adult flies for 1 h at 37°C, and these flies were used for infection one day after a heat shock. *W^1118^* was used as a wild type stock and *P*[*ry*+Δ2–3]*sb*/*TM6B*, *TB* was used as a genomic transposase source. The *Imd* and *spz^rm7^* were a gift from Dr. Won-Jae Lee, and *Dif ^2^* was a gift from Dr. Kwang-Min Choe.

### Infection and survival experiments

*Beauveria bassiana* from three day cultures (per 1.0 L distilled water: Dextrose 10 g, Peptone 2.5 g, Yeast extract 5 g, 25°C). *Staphylococcus aureus* (per 1.0 L distilled water: Trypticase soy broth 30 g, 37°C), *Micrococcus luteus* and *Erwinia carotovora carotovar-15* (per 1.0 L distilled water: Beef extract 3.0 g, Peptone 5.0 g pH 6.8, 30°C) from overnight cultures were recovered by centrifuging at 6,000 rpm for 10 min at 25°C. The supernatants were discarded and the pellets were resuspended in corresponding fresh culture media. Septic injury was performed by pricking the leg disc of adult flies with a tungsten needle previously dipped into a concentrated *B. bassiana* or by injecting diluted bacteria (OD = 0.1, 55 nl) into the ventral lateral side with a thin needle using a Picospritzer III injector (Parker Hannifin, USA). Natural infections with *B. bassiana* were performed by shaking anesthetized flies for 60 sec in a Petri dish containing a sporulating fungal culture [7]. Survival rates of flies after pathogen infection were measured under identical conditions for each genotype tested. Groups of 30 adults, aged 2–4 days, were septically injured, maintained at 25°C, and transferred to a fresh vial every three days. Fewer than five percent of the total flies tested died within three hours after infection and these flies were not considered in the analyses.

### *P*-element excision

Revertants for each *P*-element insertion mutant were generated through precise excision of the *P*-element by crossing with flies containing the Δ2–3 transposase, as described by Robertson et al. [48]. Excision allele identity was confirmed by PCR and direct sequencing of the excision sites.

### Preparation of genomic DNA and PCR

Approximately 10–15 adult flies were placed in a 1.5 ml centrifuge tube and frozen in liquid nitrogen for 5 min. The frozen flies were homogenized with a small pestle and genomic DNA was isolated with a G-spin™ Genomic DNA Extraction kit (Intron, Gyeonggi-do, Korea). The oligonucleotide primers used in PCR amplifications, with each sequence shown in 5′ to 3′ orientation, are described in Table S3. The standard thermal profile for PCR amplifications was 30 cycles of denaturation at 95°C for 1 min, annealing at 50°C for 1 min, and extension at 72°C for 1 min.

### Quantitative real time PCR

Adult males were challenged with live *B. bassiana* spores and incubated at 25°C for 6 h. Total RNA was isolated from 8–10 adult flies with TRIzol (Invitrogen, Carlsbad, CA) and used for cDNA synthesis with Superscript II reverse transcriptase (Invitrogen, Carlsbad, CA). Target cDNAs were measured by real time PCR using a LightCycler 480 (Roche, Basel, Switzerland). PCR reactions contained 1×SYBR Green mix (Applied Biosystems, Foster City, CA) and were analyzed with LightCycler 480 software 4 (Roche). All results were normalized to the level of *RpL32* mRNA in each sample. Primers used are shown in Table S3.

### *In vivo* phagocytosis

*In vivo* phagocytosis assays of adult flies were performed following the procedure of Elrod-Erickson et al. and Brandt et al. [28],[49]. Groups of 3–5 day-old adult males were injected with Alexa Fluor 488-labeled heat killed spores of *B. bassiana*, fluorescein conjugated *E. coli* (K-12) BioParticles, and fluorescein conjugated *S. aureus* BioParticles (1 mg/ml, 50–60 nl) (Molecular Probes, Invitrogen) on the ventral lateral side with a thin needle using a Picospritzer III injector. Flies were incubated for 1 h at 25°C to permit phagocytosis of the spores or bacteria, followed by injection of excess Trypan blue (0.4%, 220 nl) to quench extracellular fluorescence. Phagocyte ablation experiments were performed as described by Kocks et al. [50]. CML latex beads (1.0 µm diameter, Molecular Probes) were washed in PBS and concentrated in PBS to 8% solids. Beads (100 nl) were injected 24 hours before the phagocytosis test.

Phagocytosis of India ink was observed as described in Rutschmann et al. [10]. India ink carbon particles (Pébéo, Gemenos, France) (diluted 1/50 in PBS, 90 nl) were injected on the ventral lateral side with a thin needle using a Picospritzer III injector (Parker Hannifin). The phagocytosis of India ink by the sessile blood cells was observed 2 h later. Phagocytosed signals were observed under a Zeiss Axioplan 2 microscope (Zeiss). Fluorescence particles and Indian ink around the dorsal vessel was quantified from raw unaltered pictures using Image J software (NIH, Bethesda, MD). Before the software was used to count the area of particles, each image was converted to a 32-bit grey scale image and was thresholded to highlight the particles. The phagocytic index was expressed as area of the signal corresponding to the sum of the encircled areas.

### Analysis of larval hematopoiesis

Larvae were staged according to procedures described in Zettervall et al. [32]. Emptying of the gut marks the difference between early- and late-wandering third instar larvae, therefore a red household food dye was added to the food to allow visualization of the gut contents. The six homo-lethal alleles were maintained as heterozygotes balanced with either the second chromosome balancer *CyO* or with the third chromosome balancer *Ubx*. Precisely staged late-wandering third instar larvae were rinsed well in PBS (137 mM NaCl, 2.7 mM KCl, 6.7 mM Na_2_HPO_4_, and 1.5 mM KH_2_PO_4_) and blotted on Kimwipes to remove excess PBS before bleeding. The larval cuticle was ripped gently near the posterior end while submerging the larva in 20 µl PBS. The hemocytes were transferred to a Neubauer improved hemocytometer (Marienfeld) to determine plasmocyte number. To quantify crystal cells, late-wandering third instar larvae were heated at 60°C for 10 min in a water bath to induce blackening of mature crystal cells and blackened crystal cells in the last two posterior dorsal segments of third instar larvae were counted under a dissecting microscope. For melanization reactions, third instar larvae were pricked with a clean standard needle and the reaction was observed 2 h after injury. Melanization signals were quantified from raw unaltered pictures using Image Pro Plus 4.5 software (Media Cybernetics, Silver Spring, USA). The melanization index was expressed as [area]×[mean intensity] of encircled areas.

## Supporting Information

Table S1 *P*-element insertion lines used and their survival after septic infection of *B. bassiana*.
(0.26 MB DOC)

Table S2 GenExel EP lines isolated from the antifungal screen.
(0.05 MB DOC)

Table S3 Primer sequences used for PCR analyses.
(0.06 MB DOC)

Figure S1 Germinating hyphes of *B. bassiana* on dead *Drosophila*.
(0.45 MB TIF)

Figure S2 Overexpression of the disrupted genes using the Gal4-dependent promoter of the *P*-element that was inserted at the 5′ UTR of the gene in a forward orientation.
(0.63 MB TIF)

Figure S3 Melanization induced by a clean injury in the 16 mutant larvae.
(1.26 MB TIF)
