# Supplementary material for: Identification and Functional Analysis of Antifungal Immune Response Genes in Drosophila
Source: PLoS Pathog. 2008 Oct 3;4(10):e1000168. doi: 10.1371/journal.ppat.1000168 (PMC2542415; doi:10.1371/journal.ppat.1000168)
Supplement: Table S1 — P-element insertion lines used and their survival after septic infection of B. bassiana. (0.26 MB DOC) [file ppat.1000168.s001.doc]

**Table S1.** *P*-element insertion lines used and their survival after septic infection of *B. bassiana*.

| **No** | **Clone ID** | **Symbol** | **GenExel** | ***P*-element** | **Homozygote** | **Survival^2^** | | |
| --- | --- | --- | --- | --- | --- | --- | --- | --- |
|  |  |  | **ID** | **Insertion^1^** | **lethality** |  |  |  |
|  |  |  |  |  |  | **D0** | **D3** | **D6** |
|  |  | ***W^1118^*** | **wild type** |  |  | **30** | **28** | **27** |
|  |  | ***spz*** |  |  |  | **30** | **8** | **1** |
| 1 | SD04942 | *CG31037* | GE20468 | 5'UTR | Viable | 28 | 25 | 20 |
| 2 | SD05481 | *MAPk-Ak2* | GE1680 | 5'UTR | Lethal | 30 | 28 | 22 |
| 3 | LD33388 | *CG17090* | GE29453 | 5'UTR | Viable | 30 | 27 | 26 |
| 4 | LD47563 | *mbt* | GE3935 | 5'UTR | Lethal | 30 | 28 | 27 |
| 5 | LP03320 | *sktl* | GE15474 | 5'UTR | Lethal | 30 | 29 | 28 |
| 6 | LD41207 | *Dsor1* | GE8094 | CDS | Lethal | 30 | 29 | 28 |
| 7 | GH01941 | *CG8445* | GE14545 | 5'UTR | Viable | 30 | 30 | 30 |
| 8 | LD46760 | *CG1440* | GE1457 | 5'UTR | Viable | 30 | 27 | 23 |
| 9 | SD10626 | *olf186-F* | GE15261 | 5'UTR | Viable | 30 | 27 | 25 |
| 10 | LD30894 | *CG2264* | GE10107 | 5'UTR | Viable | 30 | 29 | 28 |
| 11 | AT24588 | *inv* | GE10665 | 5'UTR | Viable | 30 | 15 | 11 |
| 12 | SD07795 | *Sin3A* | GE11260 | 5'UTR | Viable | 30 | 29 | 25 |
| 13 | LD28689 | *ttk* | GE23411 | 5'UTR | Viable | 30 | 26 | 26 |
| 14 | SD01229 | *sbb* | GE13841 | 5'UTR | Viable | 30 | 22 | 21 |
| 15 | LD29371 | *da* | GE15558 | 5'UTR | Lethal | 30 | 28 | 28 |
| 16 | GM14667 | *CG3800* | GE15642 | 5'UTR | Viable | 30 | 26 | 26 |
| 17 | GH03826 | *CG12744* | GE13306 | 5'UTR | Viable | 30 | 19 | 4 |
| 18 | LD15904 | *CG7752* | GE23983 | 5'UTR | Viable | 30 | 26 | 24 |
| 19 | GH14389 | *CG32529* | GE1283 | CDS | Viable | 30 | 28 | 24 |
| 20 | SD09488 | *Pcl* | GE15295 | 5'UTR | Lethal | 30 | 17 | 7 |
| 21 | LD21880 | *CG6227* | GE4262 | CDS | Viable | 30 | 28 | 27 |
| 22 | SD01276 | *su(w[a])* | GE4328 | 5'UTR | Lethal | 30 | 30 | 30 |
| 23 | LD41427 | *CG7263* | GE14994 | CDS | Lethal | 30 | 18 | 7 |
| 24 | SD09402 | *CG7879* | GE27632 | 5'UTR | Viable | 30 | 27 | 26 |
| 25 | LD33076 | *elav* | GE8483 | 5'UTR | Lethal | 30 | 27 | 27 |
| 26 | LD10786 | *l(2)35Df* | GE16991 | CDS | Lethal | 30 | 26 | 25 |
| 27 | LD15253 | *spen* | GE10359 | 3'UTR | Viable | 30 | 7 | 2 |
| 28 | LD42739 | *Aats-glupro* | GE20332 | 5'UTR | Lethal | 30 | 27 | 27 |
| 29 | GH21710 | *CG6833* | GE24544 | 5'UTR | Viable | 30 | 26 | 25 |
| 30 | GH03108 | *CG2790* | GE10412 | CDS | Lethal | 30 | 29 | 23 |
| 31 | LD08152 | *Trc8* | GE28803 | 5'UTR | Lethal | 30 | 30 | 29 |
| 32 | LD31556 | *CG5543* | GE12862 | 5'UTR | Viable | 30 | 27 | 26 |
| 33 | LD32717 | *CG6181* | GE10187 | 5'UTR | Lethal | 30 | 19 | 14 |
| 34 | GH03482 | *CG10249* | GE12377 | CDS | Viable | 30 | 25 | 23 |
| 35 | LD29875 | *CG5742* | GE17167 | CDS | Lethal | 30 | 28 | 22 |
| 36 | HL07962 | *CG1841* | GE2680 | 5'UTR | Viable | 30 | 27 | 19 |
| 37 | GH06265 | *Prosap* | GE14702 | 5'UTR | Lethal | 30 | 26 | 24 |
| 38 | GH21134 | *CG6741* | GE10629 | 5'UTR | Viable | 30 | 28 | 21 |
| 39 | LD14270 | *Cas* | GE15792 | CDS | Lethal | 30 | 28 | 28 |
| 40 | LD32009 | *Rep2* | GE29902 | 5'UTR | Viable | 30 | 29 | 28 |
| 41 | SD08659 | *trio* | GE23171 | 5'UTR | Lethal | 30 | 30 | 28 |
| 42 | SD04969 | *CG30372* | GE12559 | 5'UTR | Viable | 30 | 25 | 19 |
| 43 | LD40005 | *CG5036* | GE16639 | 5'UTR | Viable | 30 | 29 | 23 |
| 44 | LD31046 | *CG31163* | GE21205 | 5'UTR | Viable | 30 | 25 | 22 |
| 45 | GH20028 | *CG6204* | GE31890 | CDS | Viable | 30 | 30 | 25 |
| 46 | LD45246 | *CG5916* | GE29383 | CDS | Lethal | 30 | 26 | 25 |
| 47 | LD39079 | *CG5004* | GE1276 | CDS | Viable | 30 | 29 | 25 |
| 48 | LD26789 | *Ranbp21* | GE353 | 5'UTR | Viable | 30 | 28 | 27 |
| 49 | SD07737 | *CG6860* | GE13736 | CDS | Viable | 30 | 27 | 21 |
| 50 | GH26207 | *Gef64C* | GE22464 | 5'UTR | Viable | 30 | 29 | 29 |
| 51 | LD23613 | *CycB* | GE11269 | 5'UTR | Lethal | 30 | 28 | 24 |
| 52 | LD14743 | *sqh* | GE4507 | 5'UTR | Lethal | 30 | 28 | 26 |
| 53 | LD39385 | *shot* | GE11785 | 5'UTR | Viable | 30 | 27 | 25 |
| 54 | LD29816 | *ssh* | GE21467 | 5'UTR | Lethal | 30 | 30 | 28 |
| 55 | SD07266 | *ImpL2* | GE24463 | 5'UTR | Viable | 30 | 30 | 27 |
| 56 | LD37992 | *coro* | GE15547 | 5'UTR | Viable | 30 | 28 | 10 |
| 57 | LD05347 | *Fim* | GE4612 | 5'UTR | Lethal | 30 | 26 | 25 |
| 58 | LD25556 | *Fas1* | GE32201 | 5'UTR | Lethal | 30 | 30 | 29 |
| 59 | SD01662 | *Myo31DF* | GE14771 | 5'UTR | Viable | 30 | 30 | 22 |
| 60 | LD27988 | *CG32697* | GE2477 | CDS | Lethal | 30 | 26 | 25 |
| 61 | LD47995 | *CG10444* | GE16394 | 5'UTR | Viable | 30 | 24 | 22 |
| 62 | SD08136 | *CG13610* | GE23479 | 5'UTR | Viable | 30 | 30 | 29 |
| 63 | LD39658 | *JhI-21* | GE15185 | 5'UTR | Viable | 27 | 10 | 4 |
| 64 | LD29349 | *blot* | GE22641 | 5'UTR | Viable | 30 | 29 | 28 |
| 65 | GH23040 | *CG1090* | GE28008 | 5'UTR | Lethal | 30 | 27 | 27 |
| 66 | SD08803 | *Fs(2)Ket* | GE15496 | 5'UTR | Lethal | 30 | 27 | 25 |
| 67 | SD01527 | *vib* | GE29940 | 5'UTR | Lethal | 30 | 26 | 26 |
| 68 | SD01586 | *px* | GE10466 | 5'UTR | Viable | 30 | 26 | 26 |
| 69 | GH04236 | *CG7971* | GE23564 | 5'UTR | Viable | 30 | 25 | 21 |
| 70 | GH06471 | *RpS3* | GE21971 | 5'UTR | Lethal | 30 | 25 | 21 |
| 71 | GH11142 | *CG7816* | GE20868 | 5'UTR | Viable | 30 | 28 | 28 |
| 72 | LD10287 | *CG12238* | GE1493 | 5'UTR | Lethal | 30 | 27 | 27 |
| 73 | LD19244 | *CG8677* | GE16082 | 5'UTR | Viable | 30 | 28 | 27 |
| 74 | LD23804 | *CG7946* | GE25187 | 5'UTR | Viable | 30 | 30 | 29 |
| 75 | LD33329 | *CG17383* | GE25859 | 5'UTR | Viable | 30 | 29 | 27 |
| 76 | LD43055 | *CG6325* | GE29021 | 5'UTR | Lethal | 30 | 30 | 30 |
| 77 | LP02726 | *sesB* | GE4009 | 5'UTR | Viable | 30 | 29 | 27 |
| 78 | SD09634 | *Adk2* | GE17716 | 5'UTR | Viable | 30 | 29 | 25 |
| 79 | LD35137 | *B4* | GE11847 | 5'UTR | Viable | 30 | 24 | 18 |
| 80 | LD33094 | *Liprin-alpha* | GE10464 | 5'UTR | Lethal | 30 | 28 | 24 |
| 81 | LD23788 | *GlcAT-P* | GE25459 | 5'UTR | Lethal | 30 | 26 | 20 |
| 82 | GH04201 | *Myo61F* | GE23557 | 5'UTR | Lethal | 24 | 22 | 20 |
| 83 | GM08921 | *Ntf-2* | GE8561 | 5'UTR | Lethal | 30 | 27 | 25 |
| 84 | GH02203 | *wun* | GE12763 | 5'UTR | Viable | 30 | 28 | 26 |
| 85 | GH12907 | *endoA* | GE22171 | 5'UTR | Lethal | 30 | 27 | 23 |
| 86 | SD06902 | *zfh1* | GE25783 | CDS | Lethal | 30 | 28 | 28 |
| 87 | LD22706 | *shg* | GE13814 | 5'UTR | Lethal | 30 | 21 | 14 |
| 88 | GH08607 | *loco* | GE24954 | CDS | Viable | 27 | 20 | 3 |
| 89 | LD08717 | *Prosbeta5* | GE14932 | 5'UTR | Viable | 30 | 27 | 24 |
| 90 | LD08715 | *DDB1* | GE28589 | 5'UTR | Lethal | 30 | 25 | 13 |
| 91 | LD19584 | *Dl* | GE21750 | 5'UTR | Lethal | 30 | 29 | 28 |
| 92 | GH01330 | *Mer* | GE3945 | 5'UTR | Lethal | 30 | 28 | 27 |
| 93 | SD01877 | *CG32702* | GE2696 | 5'UTR | Viable | 30 | 28 | 27 |
| 94 | GH09755 | *pcs* | GE14175 | 5'UTR | Lethal | 30 | 29 | 28 |
| 95 | GH08045 | *CG3308* | GE27064 | 5'UTR | Viable | 30 | 28 | 24 |
| 96 | GH19047 | *CG3829* | GE18501 | CDS | Lethal | 30 | 26 | 25 |
| 97 | LD23634 | *CG4119* | GE1666 | 5'UTR | Viable | 30 | 25 | 20 |
| 98 | LD13772 | *Ubc-E2H* | GE3206 | 5'UTR | Viable | 30 | 30 | 28 |
| 99 | LP11503 | *Flo-2* | GE672 | CDS | Lethal | 30 | 28 | 19 |
| 100 | LD26301 | *AGO1* | GE10983 | 5'UTR | Viable | 30 | 28 | 25 |
| 101 | LD09564 | *sqd* | GE25546 | 5'UTR | Viable | 29 | 20 | 20 |
| 102 | GM07644 | *awd* | GE24086 | CDS | Lethal | 30 | 28 | 25 |
| 103 | SD06708 | *mbc* | GE21710 | CDS | Lethal | 30 | 24 | 21 |
| 104 | LD04356 | *kuz* | GE18574 | 5'UTR | Viable | 30 | 29 | 27 |
| 105 | SD05024 | *cnk* | GE13177 | 5'UTR | Viable | 30 | 26 | 25 |
| 106 | LD22682 | *CycE* | GE15032 | 5'UTR | Viable | 30 | 24 | 24 |
| 107 | GH13327 | *spir* | GE12916 | 5'UTR | Viable | 30 | 24 | 24 |
| 108 | LD32788 | *CG17273* | GE24452 | 5'UTR | Viable | 30 | 26 | 21 |
| 109 | GH12907 | *endoA* | GE30275 | 5'UTR | Viable | 30 | 30 | 28 |
| 110 | LD29476 | *Rab14* | GE15354 | 5'UTR | Viable | 30 | 27 | 27 |
| 111 | LD24749 | *jumeaux* | GE27806 | 5'UTR | Viable | 30 | 21 | 9 |
| 112 | LD46723 | *Lmpt* | GE27535 | 5'UTR | Viable | 30 | 17 | 4 |
| 113 | SD03655 | *CG12004* | GE25730 | 5'UTR | Viable | 30 | 21 | 11 |
| 114 | LD09950 | *Mo25* | GE23606 | 5'UTR | Viable | 30 | 25 | 21 |
| 115 | LD47579 | *string* | GE20632 | 5'UTR | Viable | 30 | 29 | 26 |
| 116 | LD27581 | *Syx13* | GE28721 | 5'UTR | Viable | 30 | 28 | 25 |
| 117 | LD29131 | *ncd* | GE26827 | 5'UTR | Viable | 30 | 28 | 27 |
| 118 | LD31205 | *Cdk4* | GE13908 | 5'UTR | Viable | 30 | 26 | 22 |
| 119 | GH09086 | *Rab6* | GE13031 | 5'UTR | Lethal | 30 | 23 | 7 |
| 120 | LD45244 | *CG11242* | GE12121 | 5'UTR | Viable | 30 | 30 | 26 |
| 121 | SD03042 | *Trx-2* | GE14911 | 5'UTR | Viable | 30 | 18 | 8 |
| 122 | GH25366 | *betaggt-II* | GE11329 | 5'UTR | Viable | 30 | 28 | 23 |
| 123 | GH28534 | *CG2803* | GE11356 | 5'UTR | Viable | 30 | 28 | 28 |
| 124 | LD02310 | *CG4080* | GE30071 | 5'UTR | Viable | 30 | 29 | 29 |
| 125 | GH01941 | *CG8445* | GE14545 | 5'UTR | Viable | 30 | 29 | 24 |
| 126 | GH15213 | *CG12065* | GE1665 | 5'UTR | Viable | 30 | 27 | 22 |
| 127 | GH10173 | *NetB* | GE4922 | 5'UTR | Viable | 30 | 30 | 29 |
| 128 | GH15539 | *Ptp4E* | GE5196 | 5'UTR | Viable | 30 | 30 | 28 |
| 129 | LD23686 | *Dap160* | GE12746 | 5'UTR | Viable | 30 | 30 | 30 |
| 130 | LD28549 | *CG10973* | GE20174 | 5'UTR | Viable | 30 | 28 | 26 |

**^1^:** The location of the *P*-element in the targeted gene is indicated.

^2^: The number of flies survived 3 hr (D0), 3 days (D3), or 6 days (D6) after *B. bassiana* infection is shown.
