# Supplementary material for: Identification and Functional Analysis of Antifungal Immune Response Genes in Drosophila
Source: PLoS Pathog. 2008 Oct 3;4(10):e1000168. doi: 10.1371/journal.ppat.1000168 (PMC2542415; doi:10.1371/journal.ppat.1000168)
Supplement: Table S2 — GenExel EP lines isolated from the antifungal screen. (0.05 MB DOC) [file ppat.1000168.s002.doc]

**Table S2.** GenExel EP lines isolated from the antifungal screen.

| Mutants^1^ | *P* element^2^ | Orientation^3^ | Homozygote^4^ |
| --- | --- | --- | --- |
| *spen* | 3'UTR | Forward | Viable |
| *Pcl* | 5'UTR | Forward | Lethal |
| *CG12744* | 5'UTR | Forward | Viable |
| *jumeaux* | 5'UTR | Forward | Viable |
| *inv* | 5'UTR | Forward | Viable |
| *Lmpt* | 5'UTR | Forward | Viable |
| *Trx-2* | 5'UTR | Forward | Viable |
| *DDB1* | 5'UTR | Forward | Lethal |
| *coro* | 5'UTR | Reverese | Viable |
| *shg* | 5'UTR | Reverese | Lethal |
| *loco* | CDS | Reverese | Viable |
| *Rab6* | 5'UTR | Reverese | Lethal |
| *CG12004* | 5'UTR | Forward | Viable |
| *JhI-21* | 5'UTR | Reverese | Viable |
| *CG6181* | 5'UTR | Reverese | Lethal |
| *CG7263* | CDS | Forward | Lethal |

^1^: Mutants used for the rescue experiments are marked with a shade.

^2^: Location of the *P*-element insertion in the targeted genes.

^3^: Orientation of *P-*element relative to the direction of transcription of the disrupted genes.

^4^: Lethality of the homozygote mutant flies is indicated.
