# Supplementary material for: Identification and Functional Analysis of Antifungal Immune Response Genes in Drosophila
Source: PLoS Pathog. 2008 Oct 3;4(10):e1000168. doi: 10.1371/journal.ppat.1000168 (PMC2542415; doi:10.1371/journal.ppat.1000168)
Supplement: Table S3 — Primer sequences used for PCR analyses. (0.06 MB DOC) [file ppat.1000168.s003.doc]

**Table S3.** Primer sequences used for PCR analyses

| **Target gene** | **Forward (5' to 3')** | **Reverse (5'to 3')** |
| --- | --- | --- |
| *spen* | ATACAGGGCCTAGCATAGACC | AAACTCTGGTGCAGAGTGAAAT |
| *Pcl* | AAGGCGCCAGATTAATGA | TGTGTGTGCGTGCTAGATG |
| *CG12744* | CTTGTAACCGAATGGAACACTT | AGTTGCACTTCAGCCAACG |
| *jumeaux* | TTATCATTGCTCCGCGACT | GCGAAGATTTCACCGGAA |
| *inv* | GCTGATTGGATGTTCGAAAA | GTTAATCCGCTCGTCCGT |
| *Lmpt* | TTAGTTTGCGCGGCAA | CGCATGCAAGAACCCAA |
| *Trx-2* | CCTCCTACCGGTAAGAAACT | CAAAATGAGCACGTCGC |
| *DDB1* | AGGAAACATTTTAGCGCGT | GATCCGTGGGCGAGGT |
| *coro* | GGTGAACGTGAACGCGA | GCATTCAGCGGGATTCAA |
| *shg* | TCGCCAGAAAGTACAAGTTC | GGCACTCTCTTTCTCCGTT |
| *loco* | ACCTGGCTTCCAGCGAA | CATCTATTGATTCTACGTCTCG |
| *Rab6* | TCACACAGCAACTGACTCAGA | TGCCAAAATCTCCGGAT |
| *JhI-21* | AGCCGTTTTGTTGTGTCC | CTAATGGCGAATAGCGC |
| *CG7263* | GTCACCTACTCCGCAGCCA | GTATGCCGGCGGCGA |
| *CG12004* | TTAGGTCACACACCACAACG | ATGGCCTACAGCGGTTG |
| *CG6181* | AACAGACGCTGTCCATCG | CCTGCTAAATGCACCCAG |
| *P-element* | CAATCATATCGCTGTCTCACTCA |  |
| *AttA* | AGGTTCCTTAACCTCCAATC | CATGACCAGCATTGTTGTAG |
| *CecA2* | ATTAGATAGTCATCGTGGTT | GTGTTGGTCAGCACACT |
| *Dpt* | ATGCAGTTCACCATTGCCGTC | TCCAGCTCGGTTCTGAGTTG |
| *Drom* | CTTGTTCGCCCTCTTCGCTGTC | AGCACTTCAGACTGGGGCTGCA |
| *Def* | CGCTTTTGCTCTGCTTGCTTGC | TAGGTCGCATGTGGCTCGCTTC |
| *RpL32* | AGTCGGATCGATATGCTAAGCTGT | TAACCGATGTTGGGCATCAGATACT |
