# Supplementary figures and images for: Identification and Functional Analysis of Antifungal Immune Response Genes in Drosophila
Source: PLoS Pathog. 2008 Oct 3;4(10):e1000168. doi: 10.1371/journal.ppat.1000168 (PMC2542415; doi:10.1371/journal.ppat.1000168)

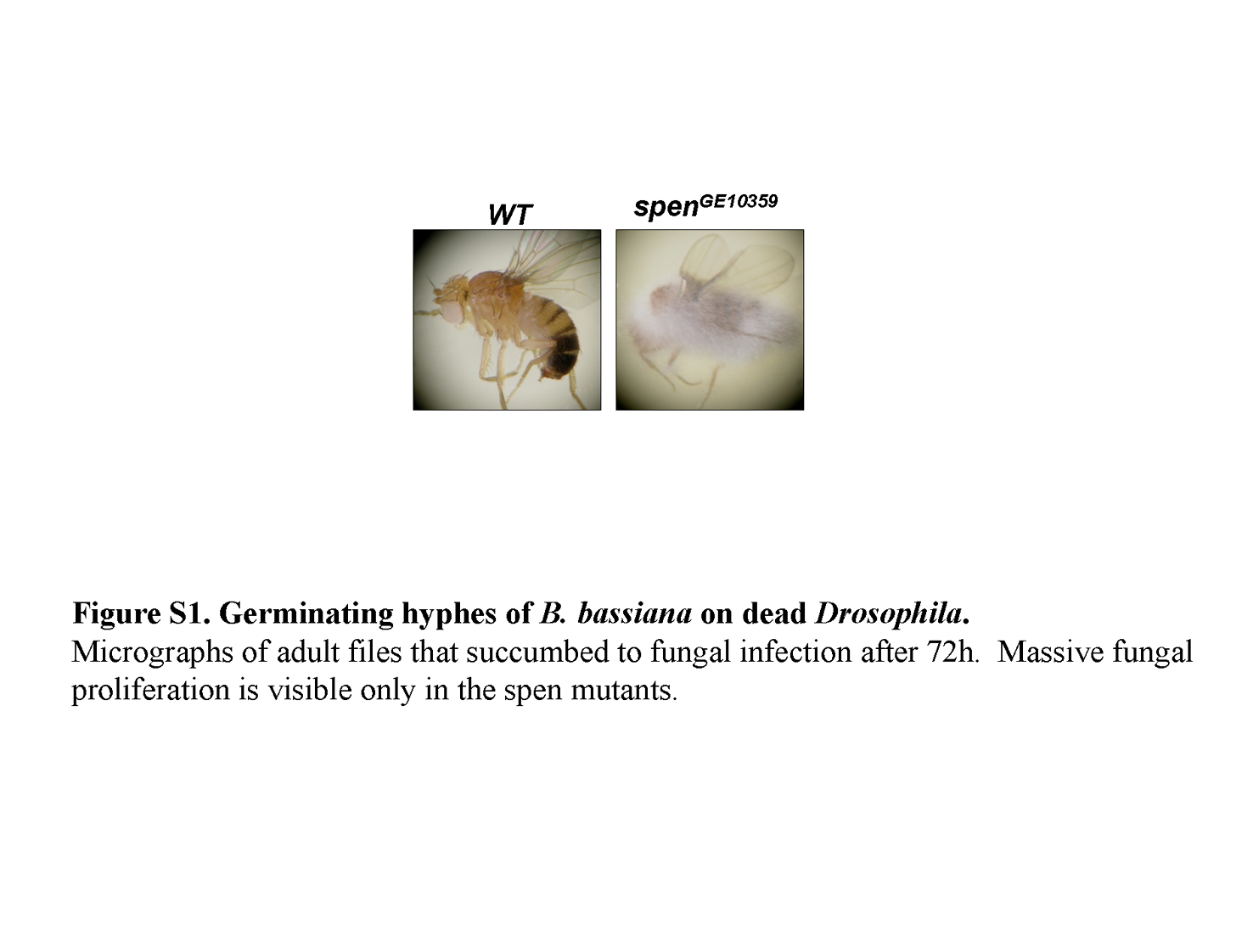

Supplement: Figure S1 — Germinating hyphes of B. bassiana on dead Drosophila. (0.45 MB TIF) [file ppat.1000168.s004.tif]

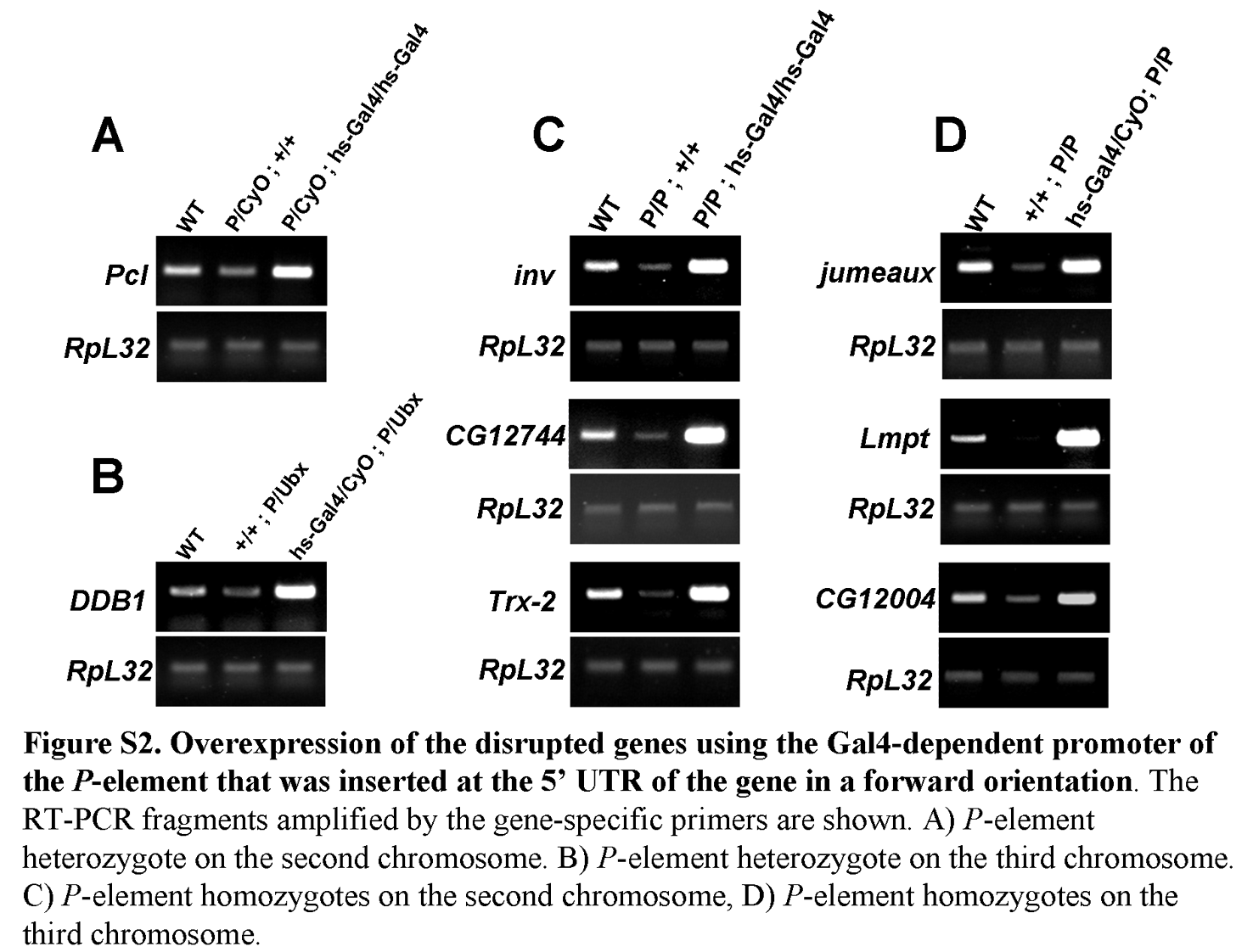

Supplement: Figure S2 — Overexpression of the disrupted genes using the Gal4-dependent promoter of the P-element that was inserted at the 5′ UTR of the gene in a forward orientation. (0.63 MB TIF) [file ppat.1000168.s005.tif]

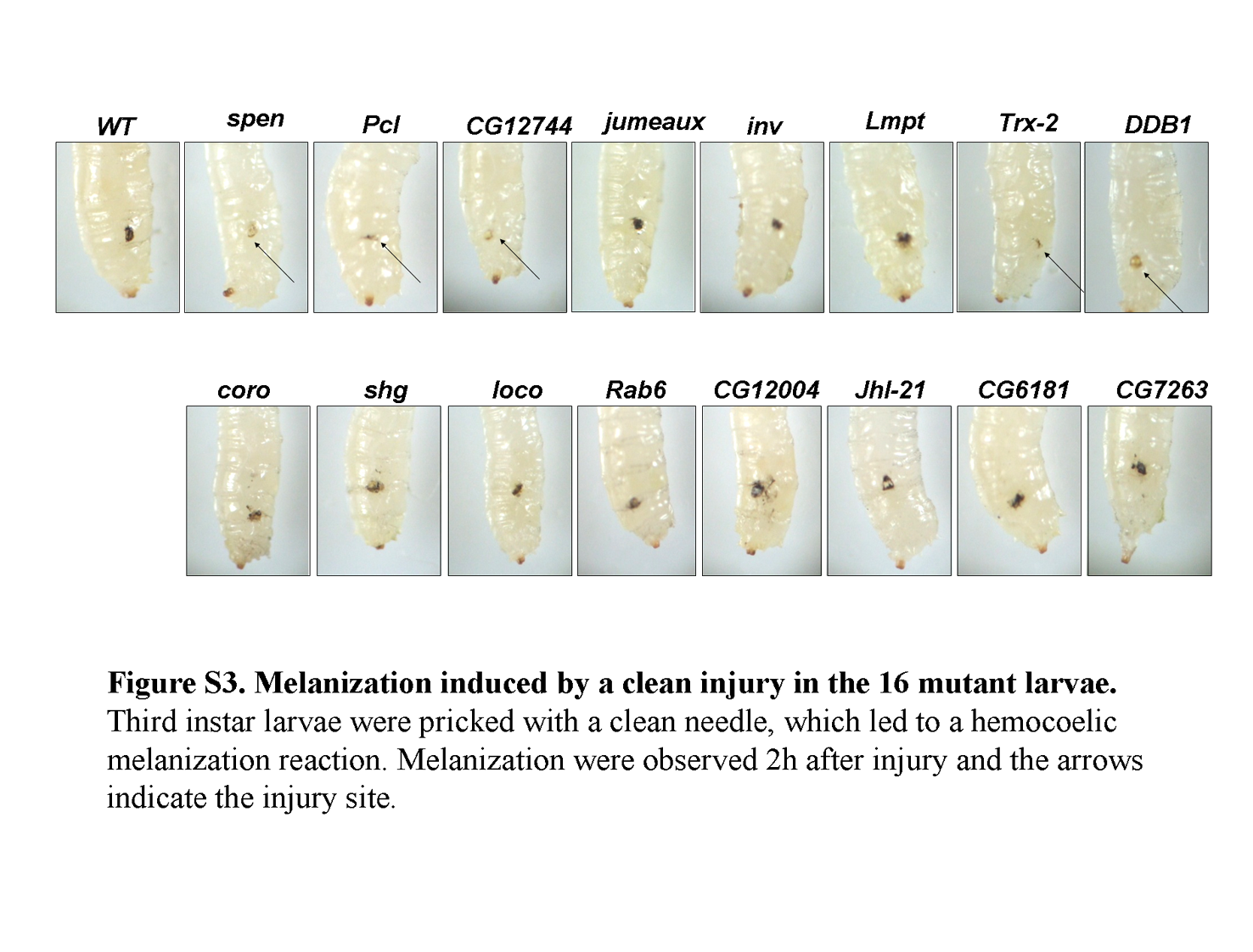

Supplement: Figure S3 — Melanization induced by a clean injury in the 16 mutant larvae. (1.26 MB TIF) [file ppat.1000168.s006.tif]
